# Effect of Urban Wastewater Discharge on the Abundance of Antibiotic Resistance Genes and Antibiotic-Resistant *Escherichia coli* in Two Italian Rivers

**DOI:** 10.3390/ijerph17186813

**Published:** 2020-09-18

**Authors:** Fabrizio Pantanella, Itziar Lekunberri, Antonella Gagliardi, Giuseppe Venuto, Alexandre Sànchez-Melsió, Massimo Fabiani, José Luis Balcázar, Serena Schippa, Maria De Giusti, Carles Borrego, Angelo Solimini

**Affiliations:** 1 Department of Public Health and Infectious Diseases, University of Rome “La Sapienza”, Piazza A. Moro 5, 00185 Rome, Italy; fabrizio.pantanella@uniroma1.it (F.P.); antonella.gagliardi@uniroma1.it (A.G.); giuseppe.venuto@uniroma1.it (G.V.); massimo.fabiani@uniroma1.it (M.F.); serena.schippa@uniroma1.it (S.S.); maria.degiusti@uniroma1.it (M.D.G.); 2 Catalan Institute for Water Research (ICRA), Scientific and Technological Park of the University of Girona, 17003 Girona, Spain; itzileku@gmail.com (I.L.); asanchez@icra.cat (A.S.-M.); jlbalcazar@icra.cat (J.L.B.); cborrego@icra.cat (C.B.); 3 Group of Molecular Microbial Ecology, Institute of Aquatic Ecology, University of Girona, 17001 Girona, Spain

**Keywords:** antibiotic resistance, antibiotic resistance genes, *Escherichia coli*, Arrone River, Tiber River

## Abstract

Background: Wastewater treatment plants (WWTPs) are microbial factories aimed to reduce the amount of nutrients and pathogenic microorganisms in the treated wastewater before its discharge into the environment. We studied the impact of urban WWTP effluents on the abundance of antibiotic resistance genes (ARGs) and antibiotic-resistant *Escherichia coli* (AR-*E. coli*) in the last stretch of two rivers (Arrone and Tiber) in Central Italy that differ in size and flow volume. Methods: Water samples were collected in three seasons upstream and downstream of the WWTP, at the WWTP outlet, and at sea sites near the river mouth, and analyzed for the abundance of ARGs by qPCR and AR-*E. coli* using cultivation followed by disk diffusion assays. Results: For all studied genes (*16S rRNA*, *intI1*, *sul1*, *ermB*, *blaTEM*, *tetW* and *qnrS*), absolute concentrations were significantly higher in the Tiber than in the Arrone at all sampling sites, despite their collection date, but the prevalence of target ARGs within bacterial communities in both rivers was similar. The absolute concentrations of most ARGs were also generally higher in the WWTP effluent with median levels between log 4 and log 6 copies per ml but did not show differences along the studied stretches of rivers. Statistically significant site effect was found for *E. coli* phenotypic resistance to tetracycline and ciprofloxacin in the Arrone but not in the Tiber. Conclusions: In both rivers, diffuse or point pollution sources other than the studied WWTP effluents may account for the observed resistance pattern, although the Arrone appears as more sensitive to the wastewater impact considering its lower flow volume.

## 1. Introduction

Antibiotic resistance (AR) has become a growing and major threat to global health, because bacteria that cause infections are becoming resistant to the most commonly prescribed antibiotics, compromising the effectiveness of antimicrobial therapy [1]. According to recent data from the European Centre for Disease Prevention and Control, over 33,000 European citizens die each year as a direct consequence of antibiotic-resistant infections, and the annual costs to the healthcare system are estimated to be EUR 1.1 billion in the European Economic Area (https://www.ecdc.europa.eu/). An in-depth understanding of the mechanisms and factors that promote antibiotic resistance is therefore urgently needed to implement cost-effective and sustainable interventions at all levels (e.g., local, regional, national, and international), as well as at the human–animal–environment interface, according to the One Health approach [2,3]. Aquatic ecosystems are not exempt from the impact of this phenomenon. In fact, the emergence and spread of AR in these ecosystems have been accelerated due to the selective pressure exerted by both natural factors and anthropogenic activities. Specifically, rivers are constantly exposed to a wide variety of chemical and biological contaminants, such as antibiotic residues and antibiotic-resistant bacteria (ARB) from anthropogenic sources (see reviews by [4,5]). Among them, urban wastewater treatment plants (WWTPs) are well-known reservoirs of many pathogenic and antibiotic resistance genes (ARGs), increasing the risk of pollution with antibiotic-resistant pathogens after effluent discharge into surface waters. Additionally, WWTPs are also hot spots for the selection and spread of ARB due to their high bacterial load and high nutrient content, which provide ideal selective and ecological conditions for horizontal transfer of genes among bacterial species [6,7]. Several factors, however, contribute both to the prevalence of ARB and ARGs in the receiving environment and to their effects on both ecosystem and public health [8,9,10]. Those include: (i) the concentration of ARB and ARGs released with the WWTP effluent; (ii) the resident river microbiota that may also be influenced by anthropogenic activities in the watershed (e.g., livestock and agricultural runoff); and (iii) the concentration of antibiotic-resistant pathogens to which river users might be eventually exposed. However, whether anthropogenic pollution in river catchment increases the abundance of ARB and indirectly causes the transfer of ARGs to human pathogens is still an open question.

The aim of this study was to assess the effects of WWTP discharges on the abundance of ARGs and antibiotic-resistant *Escherichia coli* (AR-*E. coli*) in the Arrone and Tiber rivers, differing in size and flow volume but both receiving treated effluents of urban origin into their last stretch (e.g., within 2 km from the river end in the Mediterranean Sea).

## 2. Materials and Methods

### 2.1. Study Area and Water Sampling

Sampling was conducted in the Arrone and Tiber rivers, both located near the city of Rome and both flowing into the Mediterranean Sea at ~20 km of distance from each other. The Arrone is a small river (mean annual discharge < 1 m^3^ s^−1^) draining a rural area with nonintensive agriculture and diffuse pasture land and a small town (Fregene) in its last stretch. The Tiber is a large river (mean annual discharge = 240 m^3^ s^−1^) that drains in its last stretch the urban area of Rome. In both rivers, samples were collected at different sites in the last stretch, before and after the discharge points of WWTPs located in each system (see Supplementary Table S1 for geographical coordinates of sampling sites). The WWTP discharging into the Arrone collects sewage from the town of Fregene and treats wastewater of up to 76,000 population equivalent (PE) in summer with an average discharge of 0.13 m^3^ s^−1^. In the Tiber, the WWTP collects the wastewater of the urban area of Ostia (350,000 PE and average discharge of 0.7 m^3^ s^−1^). In each river, we collected water samples ~1 km upstream (UP) of the WWTP discharge point, at the WWTP effluent (WWTP), and ~1 km downstream (DW) of the WWTP discharge point. Seawater samples were also collected at the vicinity of the river mouth to assess the impact of river pollution at the sea shore (SEA) because recreational beaches (receiving up to 300,000 beachgoers during the summer season) are located near both river mouths. Water samples were collected in triplicate using sterile, 1000 mL borosilicate bottles at the four sites on three dates (September 2016, December 2016, April 2017). River water samples were collected 1 m from the shore and at a depth of about 0.5 m with a telescopic water sampler. Seawater samples were collected at 5–10 m from the shoreline with the same procedure as river samples. Collected samples were placed in portable coolers (0 °C) and transported to the laboratory for processing within 3 h from collection.

### 2.2. Quantification of ARGs in Water Samples

From each water sample, 200 mL was filtered through 0.2-μm polycarbonate membranes and frozen for later quantification of ARGs by quantitative PCR (qPCR). Extraction of DNA from filtered samples as well as quantification of genes encoding resistance to beta-lactams (*bla*_TEM_), fluoroquinolones (*qnrS*), sulfonamides (*sul1*), tetracyclines (*tetW*), and macrolides (*ermB*) were done as previously described (Subirats et al., 2017). Also, copy numbers of the Class-1 integron-integrase gene (*intI1*) and bacterial 16S rRNA gene were quantified as a proxy for anthropogenic pollution [11] and as a proxy for bacterial abundance, respectively. Copy numbers of ARGs were further normalized to 16S rRNA gene copies to correct for sample bias due to different bacterial abundance. qPCR standard curves were obtained by analyzing 10-fold serial dilutions of DNA extracts containing known concentrations of the cloned target gene ranging from 10^8^ to 10^2^ gene copies per µL [12]. All qPCR assays were performed using an MX3005 system (Agilent Technologies; Santa Clara, CA, USA), as previously described [13]. Briefly, qPCR reactions were prepared in a final volume of 30 µL containing either SYBR^®^ Green Master Mix (Applied Biosystems) for *bla*_TEM_ or 2x Brilliant III UltraFast QPCR Master Mix (Stratagene, La Jolla, CA, USA) for the rest of the genes. Each reaction contained 200 nM of each forward and reverse primer and 10 ng of the DNA template. The final volume was adjusted to 30 µL by adding DNase-RNase-free water. Cycling conditions and primer sets for each gene were the same as previously described [12]. Samples were analyzed in duplicate with triplicate standard curve dilutions and negative controls (DNase-RNase-free water) included in each run. Specificity of amplification was determined by analysis of the melting curves (data not shown).

### 2.3. Quantification of Total Heterotrophic Bacteria and E. coli

The abundance of culturable *E. coli* was assessed using standard membrane filtration procedures [14]. Briefly, 10-fold serial dilutions of collected water samples were passed through 0.45-µm nitrocellulose filters and then the filters were placed on plates containing Tryptone Bile X-Glucuronide (TBX). TBX plates were incubated at 44 °C for 24 h. Between 10–30 colonies grown on TBX plates were picked and used to inoculate API-20E systems (Biomérieux) according to manufacturer instructions to confirm their identification as *E. coli*. About 10–30 *E. coli* isolates per sample were stored in Brain Heart Infusion Broth containing 40% glycerol at −80 °C for later determinations of phenotypic resistance to antibiotics.

### 2.4. Phenotypic Antibiotic Resistance of E. Coli Isolates

The resistance pattern of *E. coli* isolates to different antibiotics was tested through the disk diffusion technique following [15]. Selected antibiotics and concentrations used were: tetracycline (16 µg), imipenem (10 µg), chloramphenicol (30 µg), ciprofloxacin (5 µg), trimethoprim-sulfamethoxazole (64 µg), amoxicillin (2 µg), AUG2 (amoxicillin/clavulanic acid; 20/10 µg), gentamicin (10 µg), cefotaxime (5 µg).

### 2.5. Statistical Analyses

Our hypothesis was that the discharges of WWTP effluents alter the concentration of ARGs and AR-*E. coli* at downstream sites after the season effect has been accounted for. We did not make any specific a priori hypothesis on site and date interaction as this test has low power, given the sampling design carried out (three replicates per site and date) and the large within-site variability of ARGs and AR-*E. coli* abundances that might blur eventual between-site differences at each sampling date. We thus tested for differences between sites after controlling for sampling date using two-way ANOVA for continuous response variables (ARG concentrations) or logistic regression for presence/absence response variables (AR-*E. coli*). In the first analysis, the absolute abundance and relative abundance of targeted genes were log-transformed before analysis. Residuals were assessed for normality and homoscedasticity. Differences in the abundance of target genes between rivers were assessed using pairwise Student’s t-test after subsetting data frames by sampling site. In all tests, the significance level was set at α = 0.05. Logistic regression was used to assess the effect of site after controlling for sampling date on the odds of *E. coli* isolates being resistant (equal to the probability of being resistant / probability of not being resistant). Results are reported as odds ratio and its 95% confidence intervals using the upstream sites as reference and statistical significance assessed by Wald test. All analysis was carried out with R 4.0.1 [16].

## 3. Results

### 3.1. Concentration of Biomarker Genes and ARGs

**Figure 1:**
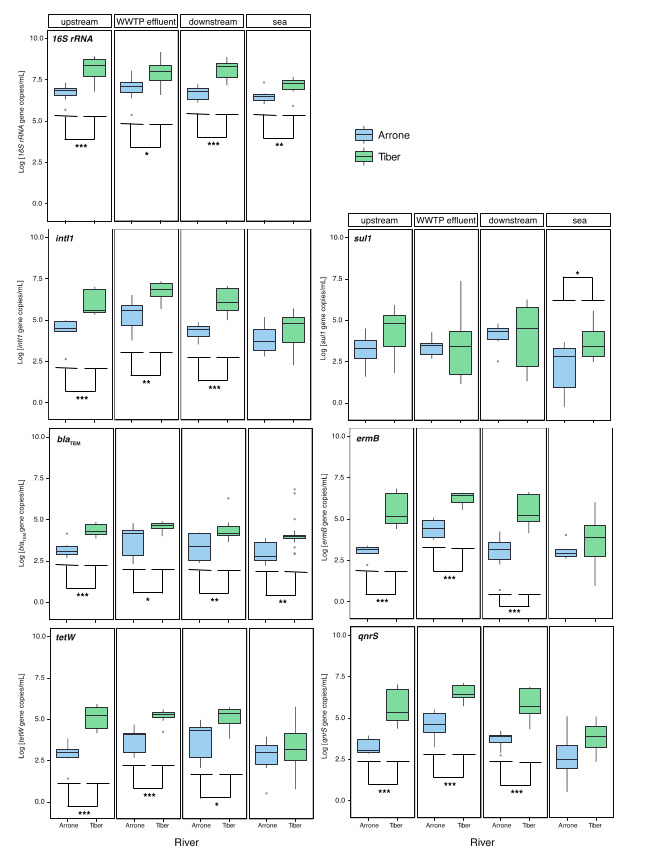
Comparison of the absolute abundance of target genes between studied rivers at the different sampling sites. The lower and upper edges of each boxplot are the first and third quartiles, the midline shows the median and the whiskers extend from the minimal to the maximal values. Outliers are indicated by gray dots. Asterisks denote statistical significance as follows: * *p* < 0.05; ** *p* < 0.01; *** *p* < 0.001.

For all studied genes, absolute concentrations were significantly higher in the Tiber than in the Arrone at all sampling sites, despite their collection date, clearly reflecting the differences between both systems (Figure 1). The only exception was the *sul1* gene, which showed no differences in abundance at all river sites but only at the sea (Figure 1). These differences in absolute abundance of ARGs between both rivers were less evident when their relative concentrations were compared (Supplementary Figure S1), and only *ermB* and *qnrS* genes showed significant differences in upstream samples collected at the Tiber. It is also relevant to point out that the prevalence of all measured genes, except the *qnrS* gene, was similar in the WWTP effluents discharging into the Arrone and the Tiber, pointing to the well-known prevalence of ARB and ARGs within WWTPs (Supplementary Figure S1).

**Figure 2:**
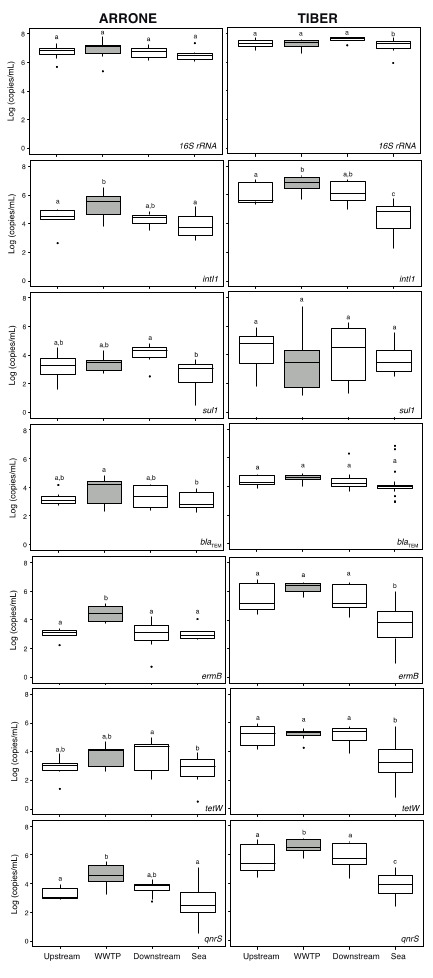
Absolute abundance of target genes in sampled sites from Arrone (left) and Tiber (right) rivers. Different letters above boxplots indicate significant differences (two-way ANOVA and post hoc Tukey test after correction of *p*-values for multiple comparisons). The lower and upper edges of each boxplot are the first and third quartiles, the midline shows the median and the whiskers extend from the minimal to the maximal values. Outliers are indicated by black dots. WWTP effluent sample is highlighted in gray.

Bacterial abundance (estimated as copy numbers of 16S rRNA genes) showed no significant differences between sampling sites in both rivers (Figure 2, top panels). Regarding the target genes, no common patterns were observed in both rivers regarding either the ARG type or the river site (Figure 2). While significant differences were measured for the *intI1* gene between upstream and WWTP effluent samples in both rivers and the *ermB* gene in the Arrone (Figure 2), most of the target ARGs did not show differences in absolute concentrations along the studied stretches. The impact of the WWTP discharges on the receiving rivers was thus too small to account for significant differences between upstream and downstream samples (Figure 2). The dilution of the river flow into the sea caused a significant decrease in the absolute abundance of ARGs, although this effect was highly dependent on the ARG type (*sul1* and *tetW* genes in the Arrone and *intI1*, *ermB*, *tetW* and *qnrS* genes in the Tiber; Figure 2). The normalization of ARG concentrations to bacterial abundance (ARG copies/16S rRNA copies) revealed similar trends, with no clear effect of effluent discharges on the prevalence of the target ARGs within the river bacterial community, even for *intI1*, *ermB,* and *qnrS* genes, which showed significantly higher prevalence in wastewater effluent samples (Supplementary Figure S2). Of particular interest was the similar prevalence of some genes (*sul1*, *bla*_TEM_, and *tetW*) across sites in both studied rivers.

### 3.2. Abundance of Escherichia Coli and Phenotypic Patterns of AR-E. Coli Isolates

Mean concentration of culturable *E. coli*, number of sampled isolates, and frequency of isolates resistant to antibiotics for each river site are shown in Table 1. In general, the concentration of *E. coli* in the Tiber was 2 to 72 times higher than in the Arrone, depending on the site (Table 1). Considering both rivers and all sites, resistance to at least one antibiotic was found in >50% of *E. coli* isolates, except for the Arrone upstream site (Table 1). Most frequent resistance of *E. coli* isolates was found for tetracycline, amoxicillin, trimethoprim-sulfamethoxazole, ciprofloxacin and chloramphenicol, while resistance to imipenem, amoxicillin, clavulanic acid, gentamicin, and cefotaxime was less frequent (Table 1).

In the Arrone, a significant site effect was found for resistance to tetracycline and ciprofloxacin, for which the odds of being resistant for an *E. coli* isolate were higher at WWTP and downstream sites compared to the upstream site (Table 2). In the Tiber, no significant increase was observed in the UP–DW comparisons for all investigated resistances (Table 2).

## 4. Discussion

Urban WWTPs are the most common pollution point sources in rivers [17] and their effects on various aspects of river ecology are well-known ([18,19,20]; among others). WWTPs are also a source of pathogens, including ARB, and drug residues, including antibiotics [21,22,23,24]. It is then not surprising that WWTPs are considered hot spots for the acquisition and spread of ARB and ARGs in the receiving water bodies [23,25,26,27]. Additionally, pathogenic *E. coli* also usually occur in WWTP effluents [28] and are enriched in rivers receiving wastewater effluent discharges [29]. Consistent with the abovementioned observations, we expected that release of ARGs and AR-*E. coli* causes an increase in the abundance of both resistance indicators at downstream sites. However, the WWTP impact on ARGs in the receiving waters was barely detectable in both rivers since both absolute and relative concentrations of target genes were similar between upstream and downstream sites, despite their higher burden in WWTP effluents. Also, when examining AR-*E. coli,* higher odds of finding an AR strain were found only for tetracycline and ciprofloxacin in the Arrone but not in the Tiber. Our results pointed to a background presence of resistant genotypes in the river, despite the collection site, that are probably caused by either diffuse or local pollution sources upstream from the WWTP discharge locations, which is not uncommon and previously observed [12]. Previous studies have demonstrated that *bla*_TEM_ and *sul1* genes are widespread among environmental bacteria, including those from locations far upstream of anthropogenic activities, as well as from pristine areas [13]. It is therefore not surprising the presence of *bla*_TEM_ and *sul1* genes as sulfonamides was the first commercially available antibiotics and that most organisms produce β-lactam antibiotics [30]. Moreover, previous studies have suggested that genes conferring resistance to tetracyclines are also widespread and their abundance (e.g., *tetW* gene) is closely related to agricultural practices [31]. These studies undoubtedly support our hypothesis because the prevalence of some genes, particularly *bla*_TEM_, *sul1,* and *tetW*, was similar across sites in both studied rivers.

When considering specifically AR-*E. coli*, it is likely that strains present in the Arrone upstream from the WWTP are those linked with agriculture/cattle runoff. Therefore, when the large microbial load of human origin enters the river with the Fregene WWTP effluent, it qualitatively increases the pattern of resistant *E. coli* by providing human-derived *E. coli* strains that were not already present in the receiving system. The whole bulk of ARGs remains, however, in similar concentration. On the contrary, the last stretch of the Tiber receives a number of small tributaries and other WWTP effluents collecting sewage from Rome, as previously observed for viruses [32], bacterial pathogens [33] and pharmaceutical compounds [34]. Therefore, pollution by AR-*E. coli* and ARGs in the Tiber was probably derived from other pollution sources located upstream of the Ostia WWTP. The ubiquitous presence of imipenem-resistant *E. coli* (mainly linked to *E. coli* of human origin) in the Tiber but not in the Arrone also supports this hypothesis.

When qualitatively analyzing the frequency of AR-*E. coli* isolates, we found high prevalence of resistance to tetracycline, amoxicillin, trimethoprim-sulfamethoxazole, ciprofloxacin, and chloramphenicol, results that are consistent with many other studies conducted in freshwater ecosystems (see the review by [35]). Interestingly our data showed that, although the *intI1* concentrations in both WWTP effluents were at least five times higher than in upstream sites, they only slightly and insignificantly increased between downstream locations. The increased availability of ARGs in WWTPs could facilitate their propagation and dissemination to the natural microbiota at downstream sites via horizontal gene transfer mechanisms. For example, it is well-known that class 1 integrons are responsible for the acquisition and spread of ARGs in Gram negative pathogens [36,37] and their increased concentration has been reported at polluted sites [38]. Accordingly, our results showed that investigated WWTPs were sources of ARGs but the differences between the upstream and the downstream sites were not detectable statistically, possibly for the same reasons outlined before. Significant increases in *intI1* and ARGs in the receiving river below the WWTP effluent were also observed by others (e.g., [39,40,41]). Nevertheless, a recent systematic review [35] found that out of 23 studies comparing the abundance of ARB between sites located upstream and downstream from a WWTP discharge, only eight studies reported a higher proportion of ARB at downstream site(s) while the remaining 14 studies reported no association or clear trend. Other explanations for the lack of statistical significance in the proportion of *intI1* between the upstream and the downstream sites include the confounding effect of other sources of pollution and seasonal variation. However, the introduction of *intI1* (and/or AR-*E. coli*) from sources other than WWTPs was unlikely because of close proximity of the upstream and the downstream sites and the lack of other inflows (like small channels or other sewages) in the studied stretch. Regarding the seasonal effect, like other studies of this kind, time-dependent variability in microbial concentrations and river discharge might decrease the statistical power to detect differences between the upstream and the downstream sites. More replicates should be collected at specific time point to exclude this bias.

## 5. Conclusions

Only recently, few investigations have quantified the risk of infection due to (accidental) ingestion of AR-*E. coli* during recreational activities in the sea [42,43]. Our data support other observations that estuaries play a natural attenuation role in inhibiting dispersal of AR-*E. coli* [44]. The observed attenuation (up to 1000 times) of the microbial concentrations in the sea can be explained with the dilution in seawater, dispersal through marine currents and winds, and/or the differential adaptability of microbial species to the marine environment. Additionally, the larger size and environmental complexity shown by the Tiber river plume [45] might explain the lower concentrations found at Tiber sea sites compared to the Arrone sea site. Accordingly, effects of human health caused by ingestion of ARB or ARGs from the sea water remain to be understood [42] and the presence of pathogenic strains should also be measured at least for microbial indicator species such as *E. coli*. Indeed, a recent analysis of the genomic diversity of *E. coli* in German WWTPs revealed that at least 180 genes might be involved in resistance mechanisms to 18 antibiotics and an average of 153–155 virulence factors per isolate [28]. Therefore, more extensive genomic analysis is needed to estimate the extent of resistance determinants for bacteria with clinical relevance present in the Arrone and Tiber.

## Figures and Tables

**Table 1:** Mean culturable *Escherichia coli* concentration (CFU/mL; standard deviation), number of sampled isolates, and frequency of isolates with phenotypic antibiotic resistance per sampling site. Trim/Sulfa: Trimethoprim + Sulfamethoxazole, Amox/Clav: Amoxicillin + Clavulanic acid. UP: upstream; WWTP (wastewater treatment plant): WWTP effluent; DW: downstream; SEA: seashore.

Parameter	Arrone				Tiber			
	UP	WWTP	DW	SEA	UP	WWTP	DW	SEA
*E. coli* mean (SD)	3.4 (2.0)	62.3 (7.7)	21.9 (6.2)	0.9 (9.9)	102 (5.4)	94.3 (3.3)	120.8 (4.3)	0.2 (3.3)
N° isolates	40	36	53	33	57	31	51	42
Tetracycline	0.13	0.33	0.38	0.30	0.40	0.19	0.25	0.31
Chloramphenicol	0.08	0.11	0.11	0.12	0.14	0.23	0.16	0.12
Ciprofloxacin	0.03	0.33	0.21	0.12	0.16	0.10	0.06	0.05
Trim/Sulfa	0.15	0.14	0.21	0.30	0.26	0.19	0.10	0.07
Amox/Clav	0.10	0.11	0.09	0.12	0.11	0.13	0.18	0.05
Imipenem	0.03	0.08	0.08	0.00	0.04	0.03	0.04	0.00
Cefotaxime	0.00	0.08	0.11	0.00	0.07	0.03	0.04	0.00
Amoxicillin	0.10	0.19	0.19	0.30	0.37	0.32	0.29	0.36
Gentamicin	0.05	0.11	0.13	0.03	0.09	0.10	0.04	0.05

**Table 2:** Differences in the frequency of antibiotic resistance pattern of *E. coli* isolates between sites after controlling for sampling date. Reported values are odds ratio (OR) and lower and upper bounds of 95% confidence intervals calculated with logistic regression using the upstream site (UP) as reference. The *p*-value of the site effect was calculated with the likelihood ratio test between models with and without the site term. The analysis for cefotaxime was not carried out because of complete separation in some of the samples. Significant *p*-values are indicated in bold type. Trim/Sulfa: Trimethoprim + Sulfamethoxazole, Amox/Clav: Amoxicillin + Clavulanic acid. UP: upstream; WWTP: WWTP effluent; DW: downstream; SEA: seashore.

	Arrone				Tiber			
Antibiotic	WWTP	DW	SEA	*p*-Value Site Effect	WWTP	DW	SEA	*p*-Value Site Effect
Tetracycline	3.6 (1.1, 12.6)	4.5 (1.6, 15)	2.8 (0.8, 10)	0.031	0.3 (0.1, 0.9)	0.5 (0.2, 1.2)	0.7 (0.3, 1.6)	0.144
Chloramphenicol	1.4 (0.3, 7.6)	1.6 (0.4, 8.3)	2 (0.4, 11)	0.846	1.9 (0.6, 5.9)	1.1 (0.4, 3.3)	0.8 (0.2, 2.6)	0.579
Ciprofloxacin	19 (3.4, 358.6)	10.7 (1.9, 201.4)	6.7 (0.9, 136)	0.002	0.5 (0.1, 2)	0.3 (0.1, 1.2)	0.3 (0, 1.2)	0.226
Trim/Sulfa	0.9 (0.2, 3.2)	1.5 (0.5, 4.7)	2.7 (0.9, 9)	0.234	0.7 (0.2, 1.9)	0.3 (0.1, 0.9)	0.2 (0, 0.7)	0.026
Amox/Clav	1.1 (0.2, 5.2)	0.9 (0.2, 4)	1.3 (0.3, 6)	0.976	1.4 (0.3, 5.5)	1.8 (0.6, 5.8)	0.4 (0.1, 2)	0.278
Imipenem	3.3 (0.4, 68.2)	3.3 (0.5, 65.8)	0.1 (0.0,70.8)	0.188	0.8 (0, 8.5)	1.2 (0.1, 10.5)	0.1 (0.0, 12.5)	0.501
Amoxicillin	2.2 (0.6, 9)	2.1 (0.6, 8.1)	4.1 (1.2, 17)	0.164	0.8 (0.3, 2.2)	0.7 (0.3, 1.6)	0.8 (0.3, 1.9)	0.857
Gentamicin	2 (0.4, 15.7)	3.3 (0.7, 23.6)	0.7 (0, 8)	0.305	1.2 (0.2, 5.6)	0.4 (0.1, 2)	0.4 (0, 1.9)	0.435

## Supplementary Materials

The following are available online at https://www.mdpi.com/1660-4601/17/18/6813/s1, Figure S1: Comparison of the relative abundance of target genes between studied rivers at the different sampling sites. The lower and upper edges of each boxplot are the first and third quartiles, the midline shows the median and the whiskers extend from the minimal to the maxi- mal values. Outliers are indicated by grey dots. Asterisks denote statistical significance as follows: * *p* < 0.05; ** *p* < 0.01; *** *p* < 0.001., Figure S2: Relative abundance of target genes in sampled sites from rivers Arrone (left) and Tiber (right). Different letters above boxplots indicate significant differences (two-way ANOVA and Post-hoc Tukey test after correction of *p*-values for multiple comparisons). The lower and upper edges of each boxplot are the first and third quartiles, the midline shows the median and the whiskers extend from the minimal to the maximal values. Outliers are indicated by black dots. WWTP effluent sample is highlighted in gray, Table S1: Geographical coordinates of sampling sites.
