# Supplementary material for: Effect of Urban Wastewater Discharge on the Abundance of Antibiotic Resistance Genes and Antibiotic-Resistant Escherichia coli in Two Italian Rivers
Source: Int J Environ Res Public Health. 2020 Sep 18;17(18):6813. doi: 10.3390/ijerph17186813 (PMC7557954; doi:10.3390/ijerph17186813)
Supplement: Supplementary file 1 [file ijerph-17-06813-s001.pdf]

Effect of urban wastewater discharge on the abundance of antibiotic resistance genes and antibiotic-resistant *Escherichia coli* in two Italian rivers

Fabrizio Pantanella, Itziar Lekunberri, Antonella Gagliardi, Giuseppe Venuto, Alexandre Sànchez-Melsió, Massimo Fabiani, José Luis Balcázar, Serena Schippa[1], Maria De Giusti[1], Carles M. Borrego, Angelo Solimini

Supplementary Information

Supplementary Figure S1

Supplementary Figure S2

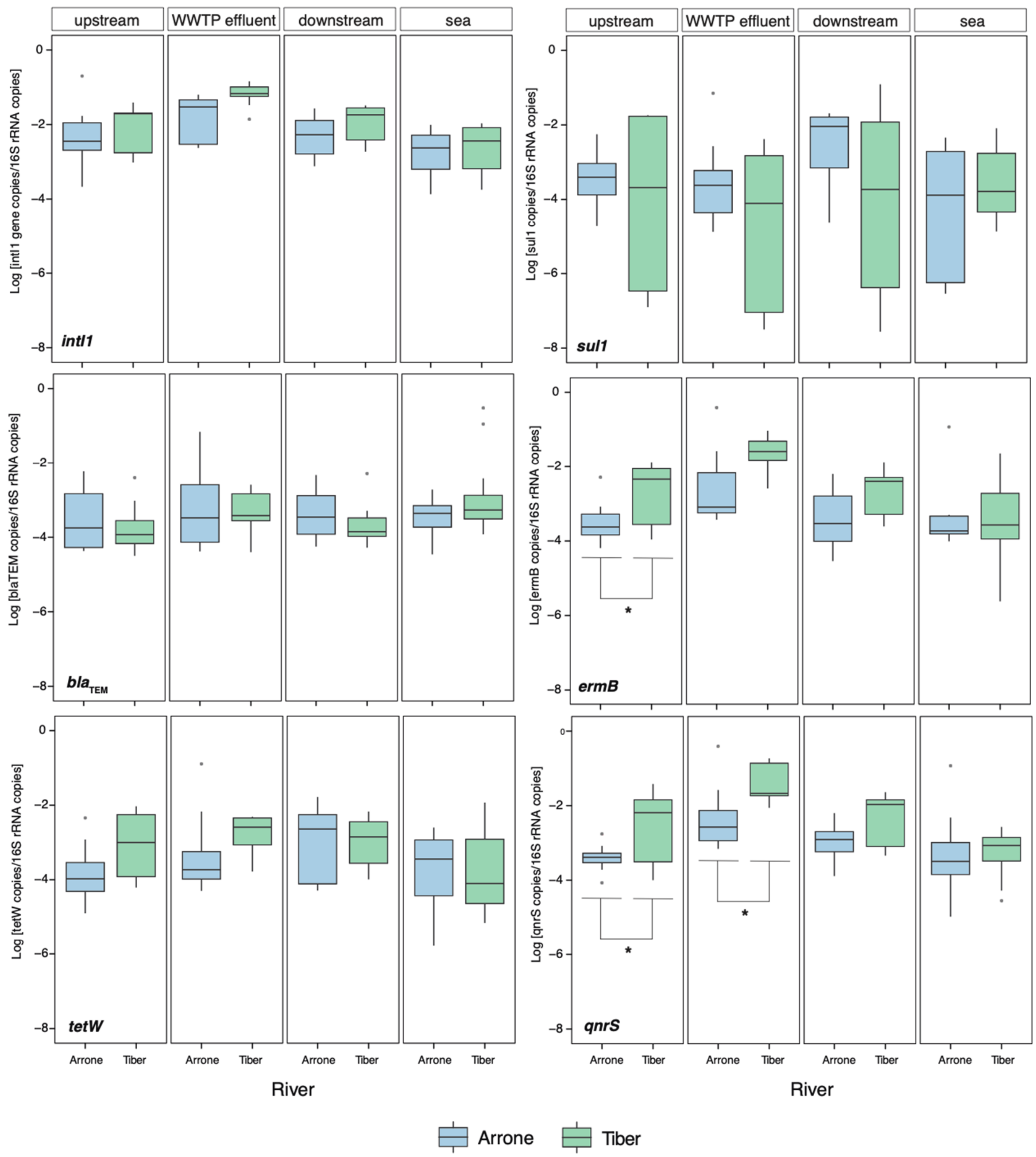

Supplementary Figure S1. Comparison of the relative abundance of target genes between studied rivers at the different sampling sites. The lower and upper edges of each boxplot are the first and third quartiles, the midline shows the median and the whiskers extend from the minimal to the maxi- mal values. Outliers are indicated by grey dots. Asterisks denote statistical significance as follows: * $p < 0.05$; ** $p < 0.01$; *** $p < 0.001$.

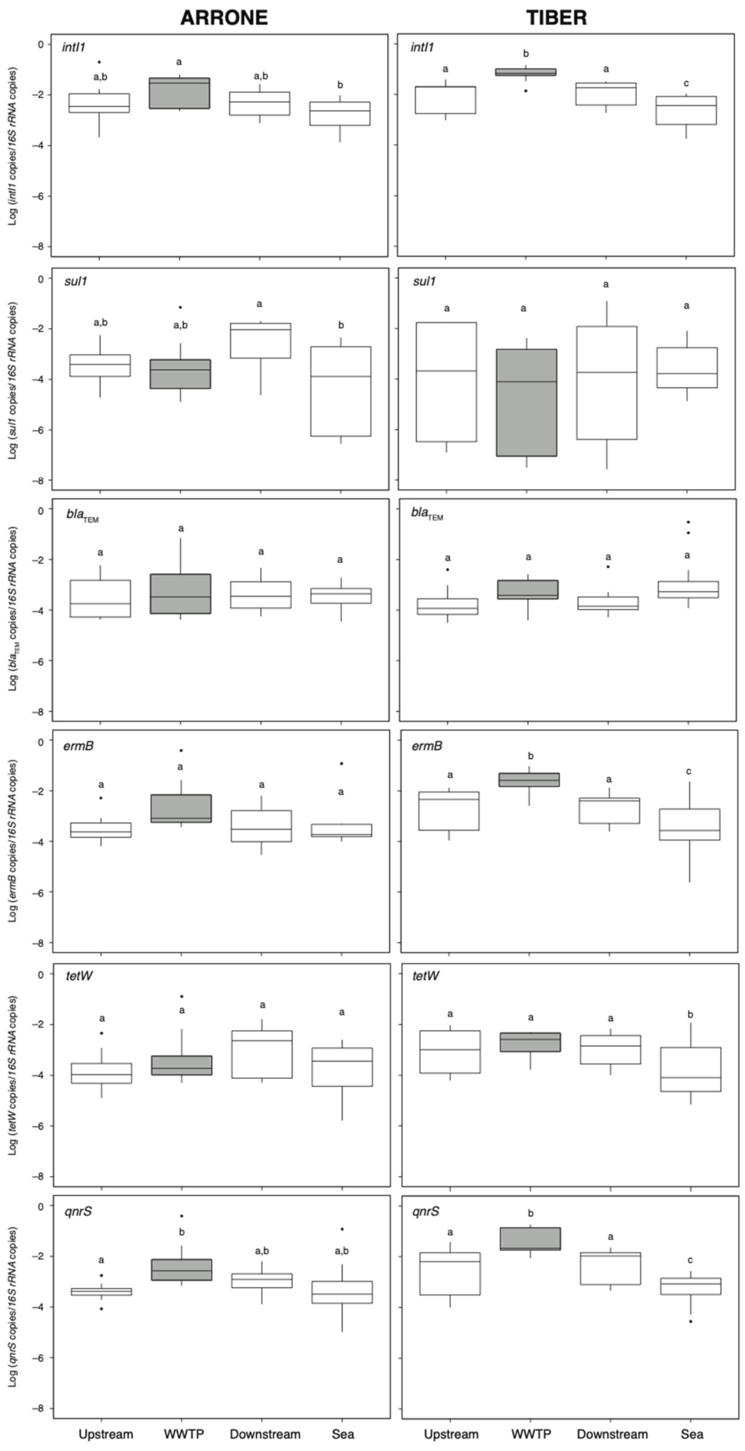

Supplementary Figure S2. Relative abundance of target genes in sampled sites from rivers Arrone (left) and Tiber (right). Different letters above boxplots indicate significant differences (two-way ANOVA and Post-hoc Tukey test after correction of *p*-values for multiple comparisons). The lower and upper edges of each boxplot are the first and third quartiles, the midline shows the median and the whiskers extend from the minimal to the maximal values. Outliers are indicated by black dots. WWTP effluent sample is highlighted in gray.

Table S1. Geographical coordinates of sampling sites

Site	Arrone	Tiber
UP	41.940297, 12.292869	41.754133, 12.277839
WWTP	41.868559, 12.189985	41.748029, 12.270138
DW	41.869421, 12.183532	41.744023, 12.251198
SEA	41.869749, 12.180023	41.736190, 12.253020
